# The Efficacy and Tolerability of Electroconvulsive Therapy in Psychiatric Patients with Arachnoid Cysts: A Retrospective Chart Study

**DOI:** 10.3390/brainsci12101393

**Published:** 2022-10-15

**Authors:** Ying Lu, Yu Tian, Yu Gan, Yixiao Fu, Qibin Chen, Lei Zou, Bangshu Zhao, Yu Yan, Shudong Liu, Xiaolu Chen, Xiao Li

**Affiliations:** 1 Department of the First Clinical Medicine, Chongqing Medical University, Chongqing 400016, China; 2 Department of Psychiatry, Chongqing Eleventh People’s, Chongqing 400038, China; 3 Department of Psychiatry, The First Affiliated Hospital of Chongqing Medical University, Chongqing 400016, China; 4 Department of Anesthesiology, The First Affiliated Hospital of Chongqing Medical University, Chongqing 400016, China; 5 Information Center, The First Affiliated Hospital of Chongqing Medical University, Chongqing 400016, China; 6 Department of Clinical Psychology II, Chongqing Mental Health Center, Chongqing 400030, China; 7 The First Branch, The First Affiliated Hospital of Chongqing Medical University, Chongqing 400015, China

**Keywords:** ECT, arachnoid cysts, psychiatry, retrospective study, depression, schizophrenia, bipolar disorder

## Abstract

Electroconvulsive therapy (ECT) is an effective therapy for many psychiatric illnesses. However, intracranial occupying lesions are a relative contraindication to ECT. Arachnoid cysts are benign, congenital, and space-occupying lesions. Our study aimed to evaluate the efficacy and tolerability of ECT in psychiatric patients with arachnoid cysts. We retrospectively identified 62 psychiatric patients with arachnoid cysts; 43 of them underwent ECT and 19 did not. Their conditions were assessed by CGI-S and different scales depending on different diagnoses (PANSS for schizophrenia; HAMD for depression; YMRS for bipolar disorder). The side effect was assessed by TESS. Significant differences were shown in the reduced scores of the CGI-S between patients who underwent ECT and those who did not (*p* = 0.001), while, at the same time, there was no significant difference in their TESS score (*p* = 0.297). The current study found that ECT is an effective and tolerable therapy for psychiatric patients with arachnoid cysts.

## 1. Introduction

Arachnoid cysts (ACs) are fluid-filled duplications or splittings of the arachnoid layer with a content which is similar but not equal to the cerebrospinal fluid [1]. Cysts may occur congenitally or secondary to head injury, intracranial hemorrhage, or infection [2]. They more usually arise in sylvian fissure (50%) [3], male (male: female = 3:1) [4] and left sidedness (left side: right side = 1.8:1) [4]. The prevalence of ACs in the population ranges from 0.8% to 2.3% [5,6,7]. Several mechanisms could account for the enlargement of these cysts: secretion by the cells forming the cyst walls, a unidirectional valve, or liquid movements secondary to pulsations of the veins [2]. Most ACs remain constant in size or become smaller [8]. They are often considered benign because more than 94% of ACs patients are asymptomatic [5]. The usual treatment recommendations are conservative but symptomatic patients with progressive hydrocephalus or intracranial hypertension are surgical candidates [1]. Treatments include cystoperitoneal (CP) shunt placement, craniotomy, endoscopic fenestration and stereotactic aspiration [9]. Above all, endoscopic fenestration can be recommended as the first-choice treatment for many ACs [1]. 

Whether arachnoid cysts are responsible for presenting symptoms is a critical question because removal of the cyst leads to relief of the symptoms, which can prove their causal relationship, while all other cases are just speculation. For instance, the cysts were often demonstrated to cause the obstruction hydrocephalus because symptoms are relieved after surgery [2]. In other case reports, arachnoid cysts were reported as a cause of increased intracranial pressure, even in non-space-occupying arachnoid cysts [10], since headache and neuro-ophthalmic signs and symptoms were relieved after a shunting procedure. In addition, arachnoid cysts were thought to be the culprit of many mental illnesses, such as schizophrenia, depression, bipolar disorder and catatonia, which were relieved after the corresponding surgical procedure [11,12,13,14,15]. However, these are only case reports.

Electroconvulsive therapy (ECT), which is an important treatment for patients with mental illness, applies an electric current to the brain to induce seizures in order to achieve a therapeutic effect. The indications for ECT go beyond major depressive disorder, mania, schizophrenia, bipolar disorder, and catatonia, and also include Parkinson’s disease, status epilepticus, self-injurious behavior in autism, obsessive compulsive disorder, and delirium [16]. Since ECT has such a wide range of applications, its safety and side effects are of even greater concern. The side effect is generally memory loss, but this has been shown to be transient [17]. Maltbie et al. [18] indicated a 74 percent overall morbidity, including a 28 percent one-month mortality rate for patients with brain tumors who received ECT and described intracranial occupying lesions as an absolute contraindication to ECT. Since then, there has been an increasing number of articles questioning the absolute nature of the contraindication and demonstrating the safe use of ECT. The literature review by Jozef Buday et al. [19] surveyed a total of 33 published and indexed case reports, case report series and a review of 75 individual patients treated with ECT in the presence of brain tumors over the last 80 years. The review indicated that, after the initial review of Maltbie et al., a growing number of case reports have shown that it is feasible to safely apply this approach to patients with benign or otherwise clinically insignificant space-occupying lesions.

Intracranial arachnoid cysts can be present as occupying lesions and have the possibility of leading to an increase in intracranial pressure [10,20]. To date, there have been few case reports on the efficacy and tolerability of ECT in psychiatric patients with arachnoid cysts [21,22,23,24,25,26,27]; this leaves very little for clinical psychiatrists to refer to when they encounter this condition, so our retrospective study aims to provide further reference by assessing the efficacy and tolerability of ECT in psychiatric patients with arachnoid cysts.

## 2. Materials and Methods

### 2.1. Participants

The current study is retrospective, and materials were extracted from inpatient clinics at the Department of Psychiatry, First Affiliated Hospital of Chongqing Medical University, China.

An extensive review of the participants’ data from (March 2014 to April 2021) was carried out. Inclusion criteria for the current study included those (1) who were diagnosed with at least one psychiatric disorder by the Diagnostic and Statistical Manual of Mental Disorders Fifth Edition (DSM-V), and were recommended ECT by psychiatrists; (2) Poor results for two or more drugs in full doses; (3) During a routine cranial CT and MRI prior to ECT, arachnoid cysts were found in the brain of these patients; (4) The patient typically had no corresponding neurosurgical presentation before. Eventually, 43 patients chose ECT after risk assessment, while 19 did not, but not necessarily because of the cysts. The number of ECT sessions was based on the patient’s clinical response. Pharmacological treatment also continued during the course of ECT. The present study protocol was approved by the Human Research and Ethics Committee of the First Affiliated Hospital of Chongqing Medical University (no. 2021-4801). 

### 2.2. Clinical Assessment

#### 2.2.1. Clinical Global Impression-Severity (CGI-S)

In the current study, CGI-S subscale was used to measure the severity of symptoms for every patient. CGI-S is rated using an 8-point scale (0–7), with no disease as 0, 1 to virtually no disease, 2 to very mild disease, 3 to mild disease, 4 for moderate illness, 5 for severe illness, 6 for severe illness and a score of 7 is given for very serious illness [28]. The reliability, sensitivity to change and utility across diagnostic groupings of this scale has been proved [29].

#### 2.2.2. Positive and Negative Syndrome Scale (PANSS)

PANSS is a scale designed to measure the severity of different types of schizophrenic symptoms. In our study, this scale aimed to evaluate the condition of schizophrenia: PANSS consists of 7 items on a positive scale and 7 items on a negative scale, with each item with a 7-point scale (from 1 to 7), with higher scores indicating more severesymptoms [30]. The reliability and validity of the Chinese vision has been proved [31].

#### 2.2.3. Hamilton Depression Scale 17 (HAMD17)

HAMD can be used to assess depressive symptoms in a wide range of disorders, including depression, bipolar disorder and neurosis, and is particularly suitable for depression. In the current study, HAMD was provided for patients with depression. The scale contains 17 entries, each on a scale of from 0 to 2 or 0 to 4, with a total score of from 0 to 52. High scores indicate high levels of depression. 0–7: no depression; 8–13: mild depression; 14–19: moderate depression; 20–25: moderate to severe depression; ≥26: severe depression [32]. The Chinese version has proven to be reliable and effective [33].

#### 2.2.4. Young Manic Rating Scale (YMRS)

YMRS is primarily used to assess manic symptoms and severity. In our study, YMRS was applied to assess bipolar severity. The scale consists of 11 items, with items 1, 2, 3, 4, 7, 10 and 11 being scored on a scale of 0–4, and items 5, 6, 8 and 9 being scored on a scale of 0–8. The total score of the scale is 60, with 0–5 being normal, 6–12 being mild, 13–19 being moderate, 20–29 being severe and 30 and above being very severe [34]. 

#### 2.2.5. Treatment Emergent Symptom Scale (TESS)

The scale [35] was used to evaluate the tolerability of psychotropic treatment and to reflect the symptoms of adverse drug reactions and laboratory changes in multiple systems. The 34 symptoms of the scale were grouped into 6 groups of symptoms: adverse behavioral reactions, laboratory tests, neurological reactions, autonomic symptoms, cardiovascular reactions, and others. A total of 1–4 points were used for each of the 34 entries. In our study, TESS was used to assess all patients for side effects.

### 2.3. Electroconvulsive Therapy (ECT)

We informed each participant and their caregivers of the benefits and side effects of ECT and obtained their written informed consent. After first three sessions performed on consecutive days, a modified bi-temporal ECT was administered every 2 days with a weekend break, using a Thymatron DGx (Somatics, LLC, Lake Bluff, IL, USA) at the First Affiliated Hospital of Chongqing Medical University. The first energy of ECT was determined based on the patient’s age: energy percentage = age × 0.7%. The stimulation energy was adjusted according to the duration of the seizure. In subsequent treatments, the energy was increased by 5% if the seizure duration was less than 25 s. Treatments continued until remission, or other reasons for stopping. The anesthetic meditation used was succinylcholine (0.5–1 mg/kg) and propofol (1.5–2 mg/kg). 

### 2.4. Pharmacological Treatment

All patients were treated by medicine. 47 patients were treated with antipsychotics, of which 21 patients were treated with olanzapine, 8 with quetiapine, 7 with amisulpride, 5 with paliperidone and 1 with risperidone. Besides, 21 patients were treated with antidepressants, of which 9 patients were treated with sertraline, 7 with Venlafaxine was used, escitalopram in four patients and mirtazapine in one patient. There is an overlap in the use of these two types of drugs. At the same time, 18 patients received other treatments, including 13 patients on benzhexol and 5 patients on valproate.

### 2.5. Statistical Analysis

Statistical analyses were conducted by the IBM Statistical Package for the Social Sciences (SPSS) version 26.0 (Armonk, New York, NY, USA, IBM Corporation). A *p*-values less than 0.05 represented statistically significant differences. Data normality was analyzed using Shapiro–Wilk test. Data that do not conform to a normal distribution were analyzed by non-parametric analysis. Student’s *t*-test was used to analyze continuous variables. χ^2^ test and Fisher’s exact test were used to analyze categorical variables. Paired *t*-tests were used to test for changes in scale scores before and after ECT. 

## 3. Results

### 3.1. Patients Characteristics

From the hospital’s data, we identified 62 psychiatric patients with a comorbidity of arachnoid cysts, 43 of whom accepted ECT and 19 of whom did not. Therefore, we divided the patients into two groups: the ECT subgroup and the No-ECT subgroup. Their clinical demographic can be seen in Table 1. For ECT subgroup, their age range was 12~49 (Mean ± SD = 25.65 ± 10.712) and the frequency of ECT ranged from 2 to 32, depending on their condition and wishes, with a Mean ± SD of 9.95 ± 7.575. The proportion of men was 51.2% and the proportion of women was 48.8%. Diagnoses among the sample were schizophrenia (n = 23, 55.8%), depression (n = 15, 34.9%) and bipolar disorder (n = 9, 9.3%) For the location of the cysts, 23 (53.5%) patients had cysts in the temporal lobe, 7 (16.3%) in the frontal lobe, 11 (25.6%) in the occipital lobe, 1 (2.3%) in the parietal lobe and 1 (2.3%) in the cerebellum. For the cerebral hemispheres of the cysts, 23 (53.5%) patients had cysts in the left side, 8 (18.6%) in the right side, 9 (20.9%) in the middle, such as the cisterna magna, and 3 (7%) patients on both sides. In addition to this, 8 (18.6%) patients had a family history (the patient’s family had at least one person who used to suffer from the same or similar mental illness). For No-ECT group, their Mean ± SD age was 28.63 ± 12.855, and a range of 14–56. 57.9% of the sample were men. The patients’ diagnoses were schizophrenia (n = 11, 57.9%), depression (n = 8, 42.1%), and bipolar disorder (n = 2, 10.5%). Cyst locations included temporal lobe (n = 15, 78.9%), frontal lobe (n = 1, 5.3%), occipital lobe (n = 2, 10.5%), and parietal lobe (n = 1, 5.3%). For the cerebral hemispheres of the cysts, 14 (73.7%) patients had cysts in the left hemisphere, 3 (15.8%) in the right hemisphere, and 2 (10.5%) in the central part. 5 (28.6%) patients had a family history. For the two groups, Student’s *t*-tests, chi-square tests and Fisher’s exact tests were carried out to determine their clinical characteristics. None of the variables described above were significantly different.

### 3.2. Clinical Profile

As mentioned earlier, different three scales were provided for patients with different disorders. Therefore, we separates data analysis for the different scales and conducted all the data analysis for TESS, CGIs and the reducing rate and score. Paired *t*-tests were used for the pre-treatment and post-treatment scale scores for both groups. Wilcoxon signed-rank test was conducted for the non-normal data (PANSS, CGI-S). There was a significant difference between pre- and post-treatment scale scores for patients who underwent ECT (*p* < 0.001, *p* < 0.001, *p* = 0.001 for PANSS, HAMD, YMRS, respectively), whereas, for patients who did not undergo ECT, there was only a significant difference for patients with depression (*p* < 0.001). Fisher’s exact tests were carried out for the two categorical variables of reduction in CGI-S scores and whether or not ECT was performed, and there was a significant difference between the patients who underwent ECT versus those that did not undergo ECT (*p* = 0.001). Student’s *t*-test was carried out to test the independence of the TESS score, and there was no significant difference in TESS scores between those who received ECT and those who did not (*p* = 0.297). See Table 2 for details.

### 3.3. Other Subgroups

In addition to seeing the impact of ECT on efficacy and side effects, many other factors should be taken into account. We divided the patients into subgroups by sex, diagnose, location of cysts, side of cysts, and family history, respectively. Student’s *t*-test and Kruskal–Wallis test were used to test the independence of the TESS scores for these subgroups. Fisher’s exact test was performed for the categorical data. See in Table 3.

### 3.4. Location and Side of Cysts

**Figure 1:**
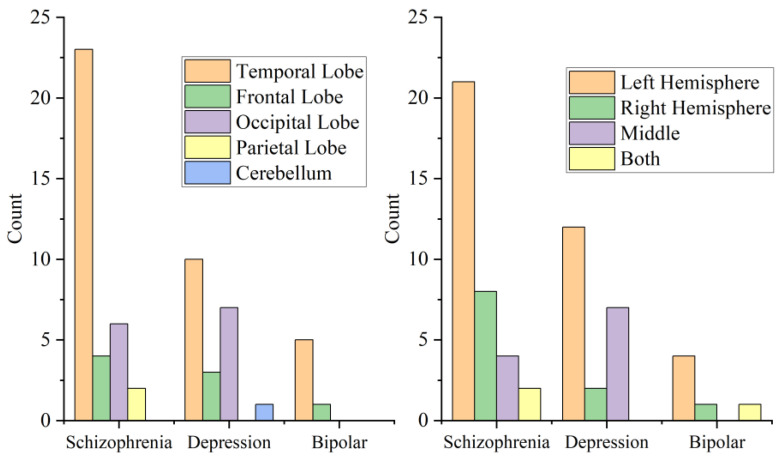
This is a figure that shows the location of brain regions or hemisphere where the cysts were located in patients with different diagnoses.

In all 62 patients, cysts were located in the temporal lobe in 38 (61.3%), in the frontal lobe in 8 (12.9%), in the occipital lobe in 13 (21.0%) and in the parietal lobe in 2 (3.2%). Cysts were located in the left hemisphere in 37 (59.7%), in the right hemisphere in 11 (17.7%), in the middle in 11 (17.7%), and 3 (4.8%) located in both sides. See Figure 1 for details. According to Fisher’s exact test, there was no significant difference between the location and side of cysts between different diseases (*p* = 0.408, *p* = 0.176, respectively). 

## 4. Discussion

In our study, there was no significant differences in post-treatment TESS scores between patients that underwent ECT and patients who did not, which means that it was tolerable for our patients with arachnoid cysts to undergo ECT. Since the introduction of ECT in 1938, there was much controversy on whether space-occupying intracranial lesions were the absolute contraindication of ECT. Some books and some of the literature mentioned this as an absolute contraindication [18,36]. In a large review of 75 patients from 33 case reports on the use of ECT with patients with brain tumor, published by Jozef Buday et al. [19], in 2020, it was proposed that ECT can be safely administered in patients with benign, small, and otherwise clinically insignificant tumors. In contrast, for arachnoid cysts, only 12 patients with arachnoid cysts were reported to safely treated with ECT and achieved remission in seven of the previous literature [21,22,23,24,25,26,27]. To the best of our knowledge, the present study is the first research on a relatively large sample of AC patients treated with ECT with tolerability. 

In this study, there was a significant difference between pre- and post-treatment scores in patients treated with ECT, whereas, in patients not treated with ECT, there was only a significant difference between pre- and post-treatment scores in patients with depression. It is also significant that patients who received ECT showed a greater decrease in CGI scores compared to those who did not receive ECT; therefore, when medication does not have the desired effect, adding ECT treatment would be a better option.

To date, all the evidence has proven that arachnoid cysts can be used in most situations with tolerability. However, according to the finding that at least some cysts may communicate surrounding cerebrospinal fluid, leading to the theoretical possibility of these cysts enlarging during the rise in intracranial pressure in the course of ECT treatments [21], and as our patients did not have neurosurgical symptoms, we still recommend neurosurgical intervention or the use of drugs (diuretics such as mannitol) to reduce intracranial pressure before ECT if the cyst is quite large.

Of the millions of people treated with ECT around the world, many had asymptomatic arachnoid cysts when there wasn’t conventional CT before ECT; they safely underwent ECT without being reported. When conventional CT was done before ECT, cases were reported that the patients were successfully treated even the arachnoid cysts were found. The absence of a single case report of death or deterioration in all searches was probably the most compelling evidence supporting the tolerability of ECT in patients with arachnoid cysts.

Are arachnoid cysts responsible for psychiatric symptoms? Arachnoid cysts were present as a cause of psychiatric symptoms in many case reports in which the patients underwent surgical procedures, and their psychiatric symptoms disappeared with the removal of cysts [11,12,13,14,15]. However, the case studies are only case studies. The only Prospective Study of Anxiety and Depression in Patients with Intracranial Arachnoid Cysts found that patients with AC had higher levels of anxiety and depression than the general population, and that anxiety and depression scores normalized after decompressive surgery [37]. However, it was not possible to determine whether these higher-than-normal anxiety and depressive symptoms were primary psychosis or cyst and pre-operative stress, and there was no control group who did not have surgery. In our study, the arachnoid cysts were discovered after the patient showed the need for electroconvulsive therapy (ECT) with psychiatric symptoms, and none of the patients required cystectomy, so we could not confirm the relationship between mental illness and arachnoid cysts.

We attempted to conduct Fisher’s exact tests between the location of the cysts, the hemisphere of the cysts and the diagnosis of our patients, and found that there was no significant difference. Cysts in all diseases were indiscriminately more frequently located in the temporal lobe and left hemisphere. Since we did not obtain data on arachnoid cysts in non-psychiatric patients, and the location of the cysts did not differ significantly among patients with different psychiatric disorders, it was not possible to conclude that psychiatric disorders are associated with arachnoid cysts in the current study. 

However, a literature search revealed temporal lobe abnormalities associated with psychiatric disorders. A review of schizophrenia-like psychoses associated with organic cerebral disorders has also been documented, where lesions in the temporal lobe and diencephalon were of particular significance [38]. In children, adolescents and elderly populations, patients with depression exhibited temporal lobe abnormalities [39,40,41]. Patients with bipolar disorder on antipsychotic medication have been reported to have abnormally enlarged temporal lobe white matter [42]. In addition, hemispheric specificity was found in depression and schizophrenia. One article showed that people with schizophrenia involves a local reduction in the grey matter of the left temporal lobe [43]. The degree of thought impairment was related to the size of the reduction in the volume of the left posterior superior temporal gyrus. In studies of left-handed people with schizophrenia [44], whose right temporal lobe showed abnormalities, it can be seen that brain pathology manifests differently in left-handed people and right-handed people with schizophrenics, and that this pathology is related to cognitive impairment. Studies of depression could reveal hyperactivity in the right hemisphere and deactivation in the left hemisphere [45]. From all these reports, we can see that psychiatric symptoms are significantly associated with hemispheric lateralisation and temporal abnormalities. Although our study could not find a relationship between arachnoid cysts and psychiatric disorders, previous case reports and studies have shown that this is still a question worthy of subsequent scholarly investigation.

The article by Steyn et al. [46] discussed the possible causes of psychiatric symptoms due to arachnoid cysts and concluded that there was plausible evidence that middle cranial fossa cysts caused neuropsychiatric symptoms. At the same time, their article explored whether psychiatric symptoms could be an indication for surgery for arachnoid cysts, and concluded that the decision for surgery should be based on a consensus between the patient, psychiatrist, and neurosurgeon. It was undeniable that there are many risks associated with neurosurgery. In some cases, neurosurgeons did not consider psychiatric symptoms to be related to cysts [47]. Even if a cyst was considered to be symptom-related, neurosurgeons did not recommend surgery because cysts were common and usually benign [48,49]. Worse, sometimes surgery could lead to complications, and even secondary psychiatric symptoms. Mace and Trimble [50] reported that six consecutive patients who had temporal lobe surgery for epilepsy, and had been referred for psychiatric assessment of psychotic symptoms. Given these risks of surgery and the fact that arachnoid cysts were usually benign and asymptomatic, these mentally ill patients with arachnoid cysts were generally routinely treated for psychiatric disorders rather than surgery. ECT is a common treatment for mental illnesses. However, this is limited by the notion that an occupying lesion is an absolute contraindication to ECT, and some clinicians had difficulty choosing whether to perform ECT in psychiatric patients with arachnoid cysts. Our article showed that psychiatric patients with arachnoid cysts were effective and tolerable, so that not only could the patient be better treated by ECT, but the risks of surgery could also be avoided.

## 5. Limitation

Several limitations of our study should be noted. Firstly, we are a retrospective study and the design was not that scientific. Secondly, our sample size was small in some subgroups. Thirdly, we did not take the size of the patient’s cyst into consideration, which may also have affected the outcomes. Additionally, we lacked data regarding a long-term follow-up for our patients. Therefore, further research is needed.

## 6. Conclusions

The current study found that ECT is an effective and tolerable therapy for patients with arachnoid cysts. ECT and drugs are more effective than drugs alone for patients with arachnoid cysts. Otherwise, the association between the location and hemisphere of the cyst and mental illness needs to be further investigated.

## Figures and Tables

**Table 1:** Patients Characteristics.

Characteristics		ECT N = 43	No-ECT N = 19	*p*
Age	Mean ± SD	25.65 ± 10.712	28.63 ± 12.855	0.541 *
	Range	12~49	14~56	
Frequency of ECT	Mean ± SD	9.95 ± 7.575	0(0)	
	Range	2~32	0(0)	
Sex	Male (%)	22(51.2)	11(57.9)	0.624 †
	Female (%)	21(48.8)	8(42.1)	
Diagnose	Schizophrenia (%)	24(55.8)	11(57.9)	1.000 †
	Depression (%)	15(34.9)	6(31.6)	
	Bipolar disorder (%)	4(9.3)	2(10.5)	
Location	Temporal Lobe (%)	23(53.5)	15(78.9)	0.284 ‡
	Frontal Lobe (%)	7(16.3)	1(5.3)	
	Occipital Lobe (%)	11(25.6)	2(10.5)	
	Parietal Lobe (%)	1(2.3)	1(5.3)	
	Cerebellar (%)	1(2.3)	0(0)	
Side	Left hemisphere (%)	23(53.5)	14(73.7)	0.491 ‡
	Right hemisphere (%)	8(18.6)	3(15.8)	
	Middle (%)	9(20.9)	2(10.5)	
	Both (%)	3(7)	0(0)	
Other chronic disease	Hypertension (%)	1(2.3)	1(5.3)	
	Diabetes (%)	1(2.3)	1(5.3)	
	Hyperlipoidemia (%)	2(4.7)	0(0)	
	lung diseases (%)	2(4.7)	1(5.3)	
Family history		8(18.6)	5(26.3)	0.513 *

* Student *t* test; † Pearson chi-square test; ‡ Fisher’s exact test.

**Table 2:** Clinical Profile.

Scale		ECT	NO-ECT	*p*
PANSS	Pre-treatment	118.96 ± 24.880	145.73 ± 20.804	0.466 *
	Post-treatment	55.67 ± 12.596	86.64 ± 26.269	<0.001 *
		***p* < 0.001 †**	*p* = 0.865 †	
HAMD	Pre-treatment	23.33 ± 3.222	24.50 ± 2.510	0.438 ‡
	Post-treatment	11.00 ± 1.813	13.17 ± 2.639	**0.042** ‡
		***p* < 0.001 §**	***p* < 0.001 §**	
YMRS	Pre-treatment	28.50 ± 4.359	35.50 ± 2.121	0.108 ‡
	Post-treatment	8.75 ± 2.062	11.50 ± 2.121	0.201 ‡
		***p* = 0.001 §**	*p* = 0.079 §	
CGIs	Pre-treatment	4.98 ± 0.801	5.63 ± 0.597	1.000 *
	Post-treatment	2.35 ± 0.482	3.53 ± 0.964	<0.001 *
		***p* < 0.001 †**	***p* < 0.001 †**	
Reducing Score of CGIs	1(%)	N = 1(2.3)	N = 7(36.8)	0.001 **
	2(%)	N = 21(48.8)	N = 4(21.1)	
	3(%)	N = 14(32.6)	N = 7(36.8)	
	4(%)	N = 7(16.3)	N = 1(5.3)	
TESS		36.44 ± 8.843	39.05 ± 9.366	0.297 ‡

* Mann–Whitney *U* test; † Wilcoxon signed-rank test; ‡ Student *t*-test; § Paired *t* test; ** Fisher’s exact test.

**Table 3:** Other factors that may affect efficacy and side effects.

		TESS Score	Reducing Score of CGIs
Sex	Male	36.21 ± 8.880	*p* = 0.765 †
	Female	38.41 ± 9.171	
		*p* = 0.341 *	
Diagnose	Schizophrenia	35.60 ± 9.708	*p* = 0.508 †
	Depression	36.95 ± 6.778	
	Bipolar disorder	47.83 ± 3.488	
		***p* = 0.006 ‡**	
Location of cyst	Temporal Lobe	38.66 ± 8.666	*p* = 0.413 †
	Frontal Lobe	39.63 ± 8.366	
	Occipital Lobe	32.00 ± 9.600	
	Parietal Lobe	34.50 ± 9.192	
	Cerebellum	38.00	
		*p* = 0.204 ‡	
Side of cyst	Left Hemisphere	37.68 ± 9.080	*p* = 0.752 †
	Right Hemisphere	37.55 ± 8.478	
	Middle	33.45 ± 9.606	
	Both	44.67 ± 2.517	
		*p* = 0.193 ‡	
Family history	YES	35.08 ± 6.525	*p* = 0.120 †
	NO	37.82 ± 9.536	
		*p* = 0.334 *	

* Student *t*-test; † Fisher’s exact test; ‡ Kruskal–Wallis test.

## Data Availability

The data will be available upon request from the Xiaolu Chen.
